# Religious teachings and sexuality of women living in Rafsanjan: A qualiattive inquiry

**Published:** 2017-12

**Authors:** Zohreh Ghorashi, Mohammad Najafi, Effat Merghati Khoei

**Affiliations:** 1 *Department of Midwifery, School of Nursing and Midwifery, Geriatric Care Research Center Rafsanjan University of Medical Sciences, Rafsanjan, Iran.*; 2 *Department of Quran Sciences and Hadith, Vali-e-Asr University of Rafsanjan, Rafsanjan, Iran.*; 3 *Brain and Spinal Cord Research Center (BASIR), Tehran University of Medical Sciences, Tehran. Iran.*

**Keywords:** Reproductive age women, Religious teaching, Sexual behavior

## Abstract

**Background:**

Islamic doctrine and related teachings play a seminal role in constructing the sexual performance of followers, women in particular**.**

**Objective:**

The aim was to explore women’s understandings of Islamic Shiite principles related to their sexuality.

**Materials and Methods:**

In a qualitative content analysis approach, four individual interviews and seven focus groups conducted in Rafsanjan, a big city in Kerman province in Iran. Content analysis was used to extract meanings and themes.

**Results:**

Three major themes were emerged describing the sexual concepts and religious-related teachings concerning women's sexual understandings and performances: “unconditional sexual submission” (Tamkin), “paradox between virtue and unconditional sexual submission” and “misconceptions”.

**Conclusion:**

Religious teachings have a basic and comprehensive role in sexuality construction and sexual health of women. However, occasional inconsistency between beliefs, learning and sexual expectations, practices, and situations would lead to jeopardize the psychological and somatic health of women. Religious-related misconceptions have essential role in creating sexual problems.

## Introduction

The scholarly debates put emphasis on the bio-psycho-socio and cultural aspects of human sexuality. Various school of thoughts theorize human sexual behaviors differently; for instance essentialists believe that sexuality is an inner essence and plays a fundamental role in humans’ sexual behaviors formation. Social constructionists believe that sexual behaviors are subjects to be learned within the society one lives (1-5). Gagnon and Simon introduced the sexual script as the basis for humans' sexual behavior rehearsal. (6).

Interaction between religion, morality, and sexuality has been revealed by various studies. Specifically The roles of religion in sexual socialization have been studied in various studies. Anarfi studied the importance of religion, and societal scripts on one's sexual socialization in a qualitative study and reported that religion was more important than the other factors in sexual socialization of women and men (7). 

In an ethnographical study conducted in Zanzibar, Beckman reported that discourses related to sexuality were coupled with the belief of being morally devoted Muslim (8). The strong relationship between religiosity and sexual conservatism has been reported by studies done with religious adolescents and youths. According to this study, low rate of high-risk behaviors and Sexually Transmitted Infections (STIs) was associated with the participants' religiosity (9-12). Other findings also support the idea that devoted religious adolescents strongly committed to sexual abstinence. They would postpone their sexual activities until marriage (13). Level of religiosity warranties sexual abstinence and somehow prevents high-risk behaviors (13). Inspiring worldwide findings, it seems both religious and sexual value systems are seminally intermingled. 

This study conducted to explore how Muslim women perceive and conceptualize the role of religion, its rules and principles on their sexual lives. This study did not examine the Islamic doctrine regarding sexual practice or the variety of interpretations. The objective of this study was to explore: 1) women’s conceptualization of Islamic Shiite principles related to sexuality; and 2) Women's sexual performances based on their understandings of religious teachings. 

We chose Rafsanjan, central city in the Kerman province, to conduct this study. In 2012, 3000 marriages and about 300 divorces were registered in the city (14). In this province people mainly practice Islam (98%). Similar to other contexts in Iran, in this province too sexual faithfulness, chastity and modesty are considered fundamental to secure family construction. Opposite sex friendship or any intimate interaction is not tolerated. Notably, access to worldwide information through social networks has trilled this construction, among women in particular. Nevertheless, still women are extremely regulated by religious principles to be submissive and follow the husband's decisions on any occasions, particularly in sexual interactions (15-17). This submission is highly appreciated and valued by devoted Muslims (18).

In this paper we tend to report meanings attached to women's understandings of Islamic Shiite principles related to their sexual lives.

## Materials and methods

Qualitative content analysis approach was employed to collect data in our mixed method study. It was aimed to explore the meanings by which women used to conceptualize their sexual behaviors and performances. Fieldwork was carried out from January 2012 to the saturation of data. 

Similar to other cities, also in Rafsanjan all women from various areas are referred to the public health centers for their children's immunization and regular child health check. Therefore, the researcher could achieve maximum variation in the purposive sampling. As our key facilitators, Midwives working in the public health centers, facilitated access to the participants, motivated them and set up the proper time to interview. At the start of each focus group discussion (FGD) session, the aim of the study was explained to all participants and a written consent form was signed by all participants. For reassuring the participants of keeping their security and privacy, they were given a pseudonym and called by these names through the sessions. 

In this project, Rafsanjan city was chosen, as the main investigator (zG) was from Rafsanjan and was a well-known and expert Midwife in this city. Inspiring Janice Morse's work, a well-known qualitative research pioneer (19), she could operate as an insider and facilitate discussing sexuality matter with the her mother town counterparts 

We conducted four individual face to face interviews to have access to our key informants. Data collection was continued by conducting seven FGDs over a 6-month's period from January to June 2012. We continued data collection till data saturation was achieved. We opened the session with the main question: “How do you define sexual behavior?” The session was carried out by molding questions based on the participants' interactions and responses.

Due to the nature of the topic, we employed FGD technique for data collection as the main data collection technique. In Iran, expression in sexuality-related topics is not anticipated. We expected that women were more comfortable when they make dialog in the group. It was anticipated that the women would motivate each other in expressing their sexual concerns and interests. In each FGD session between three to ten people participated and each session lasted between 55-80 min. After any session, based on the analyzed data, we could make the plan for next focus group discussion session. FGD sessions were carried out in urban and rural health centers. All the discussions were recorded and transcribed verbatim.

**Ethical consideration**

The project was approved by the ethics committee of Isfahan University of Medical Sciences, Isfahan, Iran. Written informed consent was obtained from all women participating in the study. 

**Statistical analysis**

Data were analyzed by content analysis according to the Graneheim approach (20). According to this approach, all transcribed texts to meaning units has been broken down, all meaning units to “condense meaning units”, and finally do extracted codes of “condense meaning units”. The emerged hypothesis was reevaluated in subsequent FGDs.

## Results

All our informants were at age of 15-49 who selected from both urban and rural areas of Rafsanjan. Out of 51 participants, 4 informants participated in individual interviews, 48 informants in focus group discussions. The mean age of participants was 35.7±8.5 yr and their average length of marriage was 14.2±8.9 yr. Participants had 2±1.4 offspring in average. The education level of participants was 38.3% high school, 14.9% primary school, and 70% were housekeeper.

Three major themes were emerged describing the sexual concepts and religious-related teachings concerning the participants' sexual understandings and performances: “unconditional sexual submission”, “occasional paradox between virtue and unconditional sexual submission”, and “misconception”.

## Discussion

In this study, we explored underpins for Women's understandings of religiosity leading their sexuality. Our findings neither claim the level of the participants' religiosity nor generalize them to all Iranian-Rafsanjani women.

**Unconditional sexual submission**

The majority of women considered “unconditional sexual submission” as their main religious duty. They defined themselves devoted Muslim if they succeed in fulfilling their sexual duties throughout their marital lives. They believed those women who use their full sexual capacities to satisfy sexual duties, are devoted Muslim. Based on the religious teachings, women believed they must be submitted themselves to the husband sexually any time husband feels sexual urge. Women's narratives revealed sense of obligation to achieve this religious goal. 

A 39 married participant put emphasis on her belief that women should be unconditionally obey their husband and accept his sexual requests" of course there is an exception such as illness or kind of disability"; "more a woman is obedient, much more she would be rewarded by God".

In the first FGD, women declared some evidence from Islamic texts “if a woman does not fulfill her husband's requests at night, the angels curse her until the morning” (1). 

The Majority of women felt guilty when they cannot or do not want to be completely obedient through their sexual interactions. The description of a faithful and pious woman who successfully attempts to satisfy God’s wishes is not very different from the description of a woman who attempts to satisfy her husband’s wishes, especially in the sexual occasions. According to this belief, God is not satisfied with a Muslim woman unless she wants to or can fully satisfy her husband’s sexual requests his full sexual satisfaction. 

Our data similar to Merghati Khoei's findings show, that religion plays a significant role in scripting the participants' sexual lives (16-18). The main script is based on the necessity of women’s full submission to their husband sexual requests, regardless of time and place. The emphasis on sexual obedience is so deeply rooted in the Islamic culture that even women who have adequately tried to be sexually obedient still may feel guilty.

“If you really believe so, God isn’t satisfied with you at all, you’re a sinner. They quote the prophet, saying that if the husband needs his wife while she is praying, then she should stop praying and answer her husband’s request. And this is how Muslim women should act" (case2, FGD5)

“We’ve been brought up with Islamic principles; God doesn’t ask women to do the housework; not just that, the woman is even entitled to receiving money in return for breastfeeding her children; but if she doesn’t fulfill her husband’s sexual wishes, that’s the worst” (case3, FGD6). 

Such feelings of guilt following sexual disobedience are particularly noticeable among first and second-generation women. This guilt, which arises from the belief in the necessity of full obedience, weakens the woman’s sexual agency and in turn threatens her mental health and welfare.

Is sexual obedience (Tamkin) a jurisprudential and legal statement and principle that is only the responsibility of women and do men have no such responsibility in the marriage contract from the perspective of Islam and Shiite?

Although books on jurisprudence and the Iranian civil law emphasize mainly the obedience of women and do not discuss the obedience of men, Allameh Helli states in Sharaya, “Noshuz (disobedience) is the failure to obey and is sometimes projected by the man and sometimes by the woman” (21). Noshuz is the jurisprudential and legal opposite of obedience and is taken to signify disobedience. 

In Tahrir al-Vasila, Imam Khomeini defines women’s noshuz as the failure to have full sexual obedience to their husband. Noshuz also presents itself when a woman does not care about her appearances and cleanliness and avoids wearing make-up at her husband’s request (22). 

The author of the well-known book Javaher al-Kalam explains, “Noshuz on the man’s part occurs when he does not respect his wife’s rights, such as ghasm (bedfellow) and nafagha (maintenance); women can claim their rights under such circumstances (23).

According to Surah An-Nisa, verse 128, “If a wife fears cruelty or desertion on her husband´s part, there is no blame on them if they arrange an amicable settlement between themselves; and such settlement is best”. The husband’s noshuz in this verse refers to his sexual reluctance and indifference toward his wife. This verse also tells women what to do to deal with their husband’s noshuz and states that improving the husband-wife relationship is prioritized over any other task. According to Allama Jafari, the husband-wife relationship can be improved by presenting to an arbitrator or a religious judge who can issue an appropriate sentence regarding the interests of both parties.

Nevertheless, women’s noshuz and its legal consequences are always discussed in detail while men’s noshuz is only addressed in brief. If man and woman have equal responsibilities toward one another and have to equally respect each others’ rights, then obedience or noshuz becomes a general subject that should concern both equally, even though one of them may have more responsibilities toward the other. Just as the woman has certain responsibilities to her husband, so does the man to his wife; and just as the woman may fail to obey, so does the man (24).

Given the discussed right of women and the subsequent responsibility of men, it appears that, not only the women participating in this study, but also many others whom the researcher has encountered in other informal settings, deem their own sexual obedience to their husband a requisite for marriage while they have never been aware of their own reciprocal rights. Most women whose sexual needs have not been met for different reasons in their marriage just learn to cope with the problems as they are or else ask for the help of a physician or consultant. However, when the husband's sexual needs are not met for any reason and regardless of the husband’s reactions, the wife is the first to bring herself to trial and proceed with condemning herself.

Religious scholars cite verse 187 of Surah Baqarah to discuss sexual rights, “It has been made permissible for you the night preceding fasting to go to your wives [for sexual relations]. They are clothing for you and you are clothing for them”. This verse removes the prohibition against sexual intercourse on Ramadan nights and introduces women as men's clothing and men as women’s clothing. In Majma al-Bayan, “being clothing for each other” has been interpreted as the peace which spouses feel in each other’s presence (25). In Tafsir al-Mizan, clothing is taken to refer to covering; that is, the spouses inhibit one another from immoral acts; they cover each other’s weaknesses and conceal each other’s nakedness (26).

Regardless of what interpretation is taken to be the right one, the verse shows the reciprocity of this right for both women and men and there is no doubt in this matter.

The mutual rights and responsibilities referred to in this verse are important because often, within a marriage contract, the civil law and public opinion consider the obedience and fulfillment of sexual needs to be the responsibility of the woman. On the verse in question, Mohammadi states, “If obedience is specific to the woman, then how the content of the verse accomplished is?” If the man is known to have no responsibilities for fulfilling his woman’s legitimate needs, how can he avoid acts of immorality? The assumption that obedience is a one-sided matter and only the woman’s responsibility is thus not only reasonable, but also rejected by God. Mohammadi raises the question of why jurisprudents and legal texts refer only to women in the discussion of sexual obedience. He further argues that, as sexual obedience is a requisite for the woman to receive her maintenance (i.e. the woman’s living expenses payable by the husband based on Islamic precepts) from the perspective of Islamic jurisprudence and since, according to many jurisprudents, sexual disobedience debars the woman from her right to maintenance, Islamic texts have only discussed woman’s obedience. However, Mohammadi holds that the widely-held belief that the woman’s right to her maintenance is subject to her sexual obedience is not in fact rooted in the Quran or in the tradition of the great Prophet of Islam (27).

Regardless of the legal foundations of the matter, the important point is the reciprocity of this responsibility and the right of both man and woman for having their sexual needs fulfilled by their spouse, although the study participants had a different attitude toward the subject and believed only in their own obligation for fulfilling their husband’s needs. If the reciprocity of this obligation is replaced with their current attitude, every woman is then free to believe that she is sometimes entitled to not be prepared for sexual interactions and that her husband may also not be prepared for such interaction at times. If this attitude prevails, the mutual right can then offset each spouse’s disobedience and therefore forego the divine punishment.

**Occasional inconsistency between virtue and unconditional sexual obedience**

The participants noted instances of inconsistency between full obedience to their husband and being virtuous. One such instance concerns fulfilling those requests of the husband that are not in harmony with religious teachings as per the woman’s opinion. The occasional inconsistency between these two religious duties tends to sometimes confuse women.

“Some women have to do a certain act and they just do it (i.e. the husband's forbidden requests). If a man wants something that the woman rejects, it is dishonorable anyways. She has to do it once. (in my opinion)If he wants to do forbidden act, he should marry someone else. Some women don’t care at all when the men come and request taboo act, but some women resist” (case4, FGD1).

According to the participants, another instance of inconsistency between full obedience and being virtuous was the necessity of performing ablution for the Morning Prayer. This may be sometimes difficult for women or is hardly possible. Based on a popular jurisprudential opinion, both sexual partners are obliged to perform ablution or wash their body entirely after every vaginal or anal sexual intercourse or after reaching orgasm. They should wash themselves before saying prayers too. These necessities become problematic in families with older children still living in the house. Under such conditions, pious women who believe in unconditional obedience face a contradiction between sacrificing a clean morning prayer with ablution for showing full obedience to their husband and sacrificing obedience for the sake of saying morning prayers in a clean state of the body.

“My daughter always notices when I take a shower. If I take a shower twice a day, she complains ‘Mom, what are you doing? You’re taking too many showers. Why are you taking showers so often? And then I have to tell her that I’m sick or, that I menstruated again and had spotting. This is how I convince her” (case5, FGD5).

“Really, it’s impossible what should I do? I know God says I should be obedient but my mother-in-law is a young widow living with us I walk to the bathroom with my towel between my legs at the end of the night many nights, it is easier to just excuse myself from my husband” (case6, interview2).

**Misconceptions**

Participants’ statements revealed their lack of knowledge regarding religious sexual duties. Most of the false beliefs were in area of what was forbidden and what was permissible in sexual matters in Islam, as there were obvious contradictions between the women’s beliefs and the actual truth of Islam.

“We called one of the maddahs (panegyrists) in the city. He said that every sexual intercourse necessitates ablution. If it was in a row, it would be makruh (abominable)” (case7, FGD1).

“They said that one can perform anal sex during menstruation. I said one is unclean anyways. I mean, the woman is unclean anyways. No matter how many times you take a shower and perform ablution, unless the menstruation ends” (case8, FGD7).

It appears that the reason for this lack of awareness and the contradictions in place is the unavailability of informed sources for guiding women in these matters as well as the shame felt when needing to ask questions about them.

According to the researchers’ knowledge and experience, some of these cases did not present real problems; however, since the participants had some false religious beliefs, they were more likely to be presented with these contradictions. The main reason for the women’s insufficient information about sexual issues, was their failure to seek correct information because of excessive shame. It should be noted, however, that the women who seek such information did not generally have access to reliable resources.

Sometimes, the women had taken advice from female maddahs with no particular religious education; other times, they had taken advice from female clergies who had not managed to solve their problem but had made it even bigger with their personal opinions just for being more cautious. Religious activists, clergies and particularly, heads of seminaries should consider these arguments and facilitate a better exchange of accurate information between enlightened and capable clergies with adequate knowledge of sexual and social issues and women presenting with sexual problems.

## Conclusion

The present study found that the participants prioritized their husband's sexual rights over their own rights. In other words, in their opinion, husband has control over their body defined by the religion and was entitled to unconditional sexual fulfillment. Common sense religious teachings have contributed to the development and reinforcement of these beliefs. Such right allows men to request sex regardless of their wife’s states and conditions and makes the wife’s disobedience subject to divine punishment.

Previous studies have also addressed the sexual behaviors developed in Iranian women because of the androcentric context in which they live, including the study by Merghati Khoei *et al* (17, 28). The present study presents the effects of living in an androcentric society on women’s conceptualization of religious instructions. In such societies, women tend to disregard their rights all at once and magnify and emphasize their responsibilities instead. Moreover, the lack of accurate information and correct religious teachings sometimes provokes these misunderstandings and therefore reduces the women’s sexual agency, capacities, and self-confidence and instead increases their anxiety when faced with different situations.

## Note

***This article extracted from Ph.D. thesis. (Zohreh Ghorashi)***
